# Contribution of Autophagy-Notch1-Mediated NLRP3 Inflammasome Activation to Chronic Inflammation and Fibrosis in Keloid Fibroblasts

**DOI:** 10.3390/ijms21218050

**Published:** 2020-10-28

**Authors:** Seongju Lee, Sun Kyeon Kim, Hyungsun Park, Yu Jin Lee, Song Hee Park, Kyung Jae Lee, Dong Geon Lee, Hoon Kang, Jung Eun Kim

**Affiliations:** 1 Department of Anatomy, College of Medicine, and Program in Biomedical Science & Engineering, Inha University, Incheon 22212, Korea; lees@inha.ac.kr (S.L.); 22182056@inha.edu (S.K.K.); hyungsun@inha.edu (H.P.); 2 Department of Dermatology, Eunpyeong St. Mary’s Hospital, College of Medicine, The Catholic University of Korea, Seoul 03312, Korea; cindyeujine1@naver.com (Y.J.L.); saccharide@hanmail.net (S.H.P.); lkj6691@hanmail.net (K.J.L.); dlcjdgnl@naver.com (D.G.L.); johnkang@catholic.ac.kr (H.K.)

**Keywords:** Inflammasome, Keloid, Notch, Autophagy

## Abstract

Keloid is a representative chronic fibroproliferative condition that occurs after tissue injury. Emerging evidence showed that activation of NACHT, LRR, and PYD domains-containing protein 3 (NLRP3) inflammasome is involved in the pro-inflammatory response in injured tissues. However, the role of NLRP3 inflammasome in keloid progression remains unclear. Notch signaling, which activates NLRP3 inflammasome, is known to contribute to scar formation in keloid, but the cause of enhanced Notch signaling in keloid is not clear. We sought to investigate whether autophagy regulates Notch1 signaling in keloid fibroblasts and determine whether Notch1 signaling might regulate NLRP3 inflammasomes and myofibroblast differentiation. An in vitro model of keloid was established by culturing primary keloid fibroblasts from patients. Expression levels of Notch1, NLRP3 inflammasome proteins, pro-inflammatory cytokines, and myofibroblast markers in keloid fibroblasts were examined and compared with those in normal fibroblasts. Autophagy known to mediate Notch1 degradation was also monitored in fibroblasts. Small interfering RNA (siRNA) targeting Notch1 was used to transfect keloid fibroblasts to further examine the role of Notch signaling in NLRP3 inflammasome activation. Expression levels of Notch1 and NLRP3 inflammasome in keloid fibroblasts increased compared to those in normal fibroblasts. Such increases were accompanied by increased LC3 levels and reduced autophagic flux. Notch1 silencing in keloid fibroblasts by siRNA transfection significantly suppressed increased levels of overall NLRP3 inflammasome complex proteins, NF-kB, and α-smooth muscle actin. Autophagy induction by rapamycin treatment in keloid fibroblasts effectively suppressed expression levels of Notch1 and NLRP3 inflammasome proteins. Decreased autophagy activity in keloid can result in Notch1-mediated myofibroblast activation and NLRP3 inflammasome signaling activation which is critical for chronic inflammation. Collectively, these results identify Notch1 as a novel activator of NLRP3 inflammasome signaling leading to chronic tissue damage and myofibroblast differentiation in keloid progression.

## 1. Introduction

After skin injury, myofibroblasts derived from activated fibroblasts proliferate and rapidly synthesize the extracellular matrix (ECM) to provide tissue integrity during repair. Fibrogenesis is self-limiting as myofibroblasts undergo senescence in normal wound healing [1]. Keloid is a chronic inflammatory scar-forming condition over the original epithelial injury where myofibroblasts continue to activate. It is aggravated by irritation and mechanical tension [1]. ECM degradation and myofibroblast senescence are deficient in keloid tissues.

Conventional treatment of keloid has relied on intralesional injection of corticosteroid, cryotherapy, or adhesive silicone compression dressing [2]. Most patients with keloid experience progression of lesions once they stop the treatment mentioned above. Some patients need to get radiation therapy to stop scar progression with a risk of developing skin cancer. Given the long-term nature of the disease, the ineffectiveness of treatment, and the possibility of adverse effects associated with the treatment, it is necessary to develop new treatment that specifically targets the pathogenesis of keloid. Keloid is seen only in human beings. It is hard to build in vitro and in vivo models of keloid. Thus, studies on the pathogenesis of keloid have been limited.

We previously reported that Notch intracellular domain (NICD) is enhanced in keloid tissues [3]. Notch signaling is known to be involved in the progress of fibrosis by acting on the upstream or the downstream of the transforming growth factor (TGF)-β pathway [4]. Notch can induce the production of TGF-β and the phosphorylation of Smad3 that favors the expression of α-smooth muscle actin (α-SMA), leading to epithelial-mesenchymal transition [5]. Notch-related molecules are overexpressed in various fibrotic disease such as scleroderma, idiopathic pulmonary fibrosis, and kidney fibrosis [5,6,7,8]. However, it is not clear whether the enhanced Notch signaling in keloid is the result of an imbalance between anabolism and catabolism [9]. 

Autophagy, a representative catabolic mechanism plays a major role to maintain the homeostasis of cells to remove damaged cells and degrade cytotoxic or unnecessary molecules [10,11]. Autophagosomes exist in the shape of a cup suitable for engulfing other intracellular substrates. When the membrane extends and fuses with lysosomes, autophagosomes degrade substrates. Beclin-1 is an initiator of autophagosomes, and LC3 plays a role in the extension and closure of the autophagosome. The cytosolic form of LC3 (LC3-I) is conjugated to phosphatidylethanolamine and form LC3-II, which is recruited to membrane of autophagosomes. Since LC3-II remains on autophagosomes until after fusion with lysosomes, the level of LC3-II reflects the number of autophagosomes and autolysosomes [12]. p62 is an autophagic receptor which binds to substrates and recruits them into the autophagosome for degradation in autolysosomes. Thus, the level of p62 inversely correlates with the activity of autophagy [12]. Autophagy impacts the survival, inflammation, and differentiation of cells. Autophagy is known to regulate Notch1 degradation, which involves in the cell fate determination and differentiation [13]. Whereas autophagy is known to contribute to cancer prevention by degrading damaged cells at first, it may aggravate tumorigenesis by down-regulating apoptosis once cancer occurs [14]. Keloid appears clinically to be in a tumor condition, but it is a benign reactive fibroproliferative condition and little is known about the role of autophagy in keloid formation. 

Inflammasome activation is induced by noxious stimuli in human keratinocytes and immune cells. NACHT, LRR, and PYD domains-containing protein 3 (NLRP3) and apoptosis-associated speck-like protein containing a CARD (ASC) can assemble in response to harmful stress and form NLRP3/ASC complex. This complex sequentially leads to maturation of procaspase-1 to caspase-1 and the release of interleukin (IL)-1β which induces cell apoptosis or inflammatory cascade [15]. Recently, it has been shown that activation of NLRP3 signaling in dermal fibroblasts can accelerate skin wound healing in mice [16]. Activation of NLRP3 inflammasome also contributes to mechanical stretch-induced endothelial-mesenchymal transition in pulmonary fibrosis [17]. The NLRP3 inflammasome can be induced in cardiac fibroblasts by inflammatory stimuli [18]. The activation of NLRP3 inflammasome and the release of IL-1β are known to accelerate tissue damage and abnormal remodeling process through NLRP3/TGF-β/Smad pathway in cardiac fibroblasts after myocardial infarction [18,19]. This suggests that improper inflammasome activation may result in persistent inflammation and tissue remodeling in keloid. However, the role of inflammasome in the pathogenesis of keloid remains unknown. 

Notch signaling in fibroblasts regulates immune infiltration of connective tissue, inflammation, and ECM remodeling. Thus, we hypothesized that increased Notch signaling in keloid might be related to decreased autophagy and NLRP3 inflammasome-mediated inflammation. To provide a basis for establishing the pathogenesis of keloid, we investigated expression levels of autophagy- and NLRP3 inflammasome-related molecules in keloid fibroblasts and determined whether Notch signaling could regulate the activation of inflammasomes and myofibroblasts in keloid fibroblasts.

## 2. Results

### 2.1. Clinical Characteristics of Patients with Keloid

Clinical characteristics of six patients with keloid are summarized in Table 1. All patients were females (age range from 18 to 65) with keloid lesions on their ear-piercing sites. All specimens were obtained from ear lobes or ear helices. This is because surgical resection is generally not preferred for the treatment of keloids in areas other than the ears, and because females do more ear piercing. Disease duration varied from 1 to 3 years. Four patients did not have any treatment before surgery. Two patients (patients 2 and 3) received intra-lesional triamcinolone injection one-month and four-month before surgery, respectively. However, their keloid gradually increased. Three patients (patients 2, 3, and 5) complained of pruritus and pain on the scar with the recent growth of keloid. Three patients showed stable lesions.

### 2.2. Notch1, α-SMA, and TGF-β3 Protein Levels are Increased in Keloid Fibroblasts

**Figure 1:**
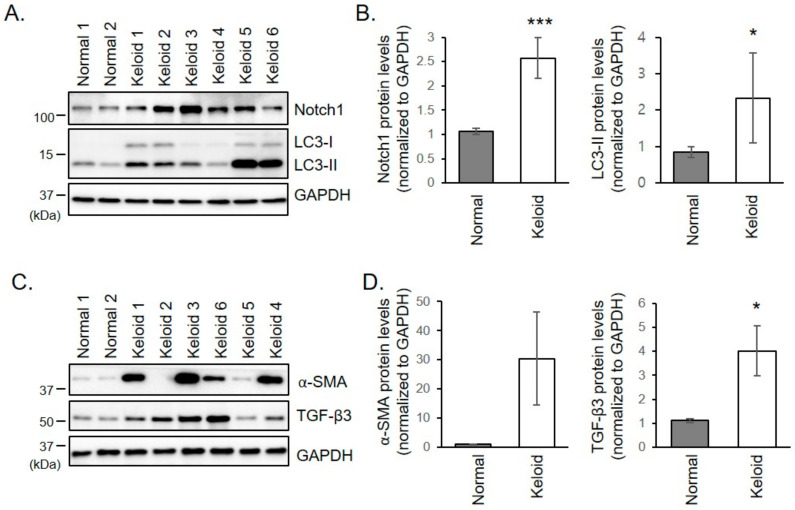
(**A**,**B**) Increased expression of Notch1 and LC3 in keloid fibroblasts. (**A**) Normal and keloid fibroblasts were subjected to immunoblotting against Notch1 and LC3. Gels are representative of five experiments. (**B**) Graphs represent means ± s.d. * *p* < 0.05; *** *p* < 0.001. (**C**,**D**) Increased expression of α-SMA and TGF-β3 in keloid fibroblasts. (**C**) Normal and keloid fibroblasts were subjected to immunoblotting against α-SMA and TGF-β3. Gels are representative of five experiments. (**D**) Graphs represent means ± s.d. * *p* < 0.05.

To investigate whether Notch signaling is involved in keloid formation, expression levels of Notch1 in keloid fibroblasts were examined. Protein levels of Notch1 were significantly increased in keloid fibroblasts than in normal fibroblasts, but the changes were variable between patients. The greater increase in Notch1 was seen in patients 2, 3, and 5, who were in the active stage with the recent growth of keloid lesions (Figure 1A,B). Keloid fibroblasts also exhibited increased expression levels of α-SMA and TGF- β3, indicating enhanced myofibroblast differentiation in keloid fibroblasts (Figure 1C,D). Whereas the expression pattern of TGF-β3 tends to correlate with Notch1, there was a great deviation between the samples in the expression of α-SMA regardless of disease activity. This discrepancy may result from individual differences in age, chronicity, and location, e.g., earlobe, and ear helix. 

### 2.3. Autophagy is Disturbed in Keloid Fibroblasts

**Figure 2:**
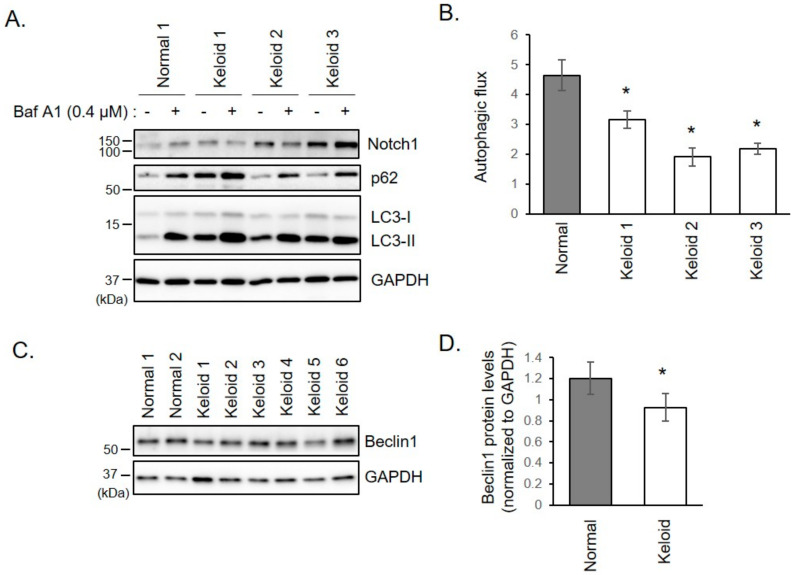
Autophagic flux is reduced in keloid fibroblasts. (**A**) Fibroblast cells were incubated with 0.4 µM of bafilomycin A1 (Baf A1). After 16 h, cells were analyzed by immunoblotting with indicated antibodies. Autophagic flux was determined by dividing the amount of LC3-II (below 15 kDa) treated with Baf A1 by the amount of LC3-II treated with DMSO. LC3-II bands were normalized to their respective GAPDH bands. (**B**) Graphs show means ± s.d. of five experiments. (**C**) Normal and keloid fibroblasts were lysed and subjected to immunoblotting using Beclin1. Gels are representative of five experiments. (**D**) Graphs show means ± s.d. of five independent experiments. * *p* < 0.05.

We investigated whether the increase of Notch1 was caused by autophagy aberration. Protein levels of LC3, an autophagosomal marker, increased in keloid fibroblasts (Figure 1A,B). The increase of LC3 represents either autophagy activation or defect of autophagosome degradation. To distinguish between these two possibilities, autophagic flux assay was performed for keloid fibroblasts derived from patients 1, 2, and 3. When autophagy status was monitored using LC3 and p62 after treatment with bafilomycin A1, an inhibitor of lysosomal degradation, the autophagic flux was reduced in keloid fibroblasts than in normal fibroblasts (Figure 2A,B). This result suggests that the increase of Notch1 level in keloid fibroblasts results from low degradation of Notch1 due to decreased autophagy activity. Beclin1, a critical autophagy initiation factor, was also down-regulated in keloid fibroblasts (Figure 2C,D). These results suggest that the increase in Notch1 in keloid fibroblasts is attributed to autophagy disturbance. 

### 2.4. NLRP3 Inflammasome Formation is Activated in Keloid Fibroblasts

**Figure 3:**
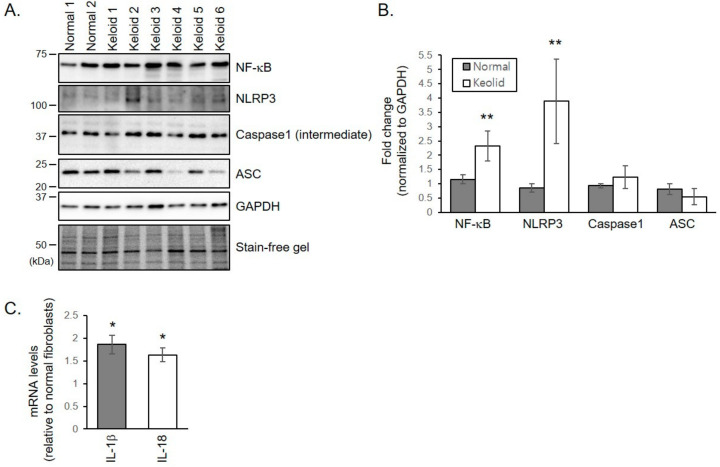
NLRP3 inflammasome formation is activated in keloid fibroblasts. (**A**) Normal and keloid fibroblasts were lysed and subjected to immunoblotting against NLRP3 inflammasome molecules. Gels are representative of five experiments. (**B**) Graphs represent means ± s.d. of five independent experiments. (**C**) mRNA levels of *IL-1β* and *IL-18* in keloid and normal fibroblasts were determined by quantitative real-time PCR. Graphs were drawn as the fold change in mRNA levels in keloids when the mRNA levels of normal fibroblasts were corrected to 1. Graphs represent means ± s.d. of three independent experiments. * *p* < 0.05; ** *p* < 0.01.

Notch signaling can promote formation of the NLRP3 inflammasome which is involved in the pro-inflammatory response in injured tissues. NRLP3 protein levels were significantly increased in keloid fibroblasts than in normal fibroblasts (Figure 3A,B). Although protein levels of ASC and an intermediate form of caspase-1 were not significantly different between keloid and normal fibroblasts, protein levels of nuclear factor kappa B (NF-κB), a known primer of the NLRP3 inflammasome, increased in keloid fibroblasts (Figure 3A,B). mRNA levels of pro-inflammatory cytokines IL-1β and IL-18 activated by the NLRP3 inflammasome were significantly increased in keloid fibroblasts than in normal fibroblasts (Figure 3C). These results indicate that NLRP3 inflammasome formation is activated in keloid fibroblasts.

### 2.5. Notch Inhibition with siRNA Down-Regulates the Activation of NLRP3 Inflammasome Formation and the Differentiation into Myofibroblasts

**Figure 4:**
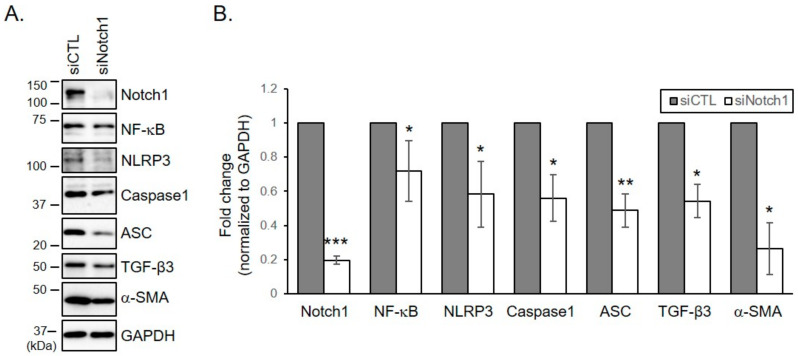
Depletion of Notch1 in keloid fibroblasts reduces increased levels of inflammasome proteins and myofibroblastic marker. Keloid fibroblasts from patients 2, 3, and 6 were transfected with indicated siRNAs (control and Notch1). After four days, cells were lysed and subjected to immunoblotting with indicated antibodies. (**A**) Gels are representative of five experiments using keloid 3. (**B**) Graphs represent means ± s.d. of five independent experiments. * *p* < 0.05; ** *p* < 0.01; *** *p* < 0.001.

To investigate whether the increase in NLRP3 inflammasome formation was caused by increased levels of Notch1 in keloid, keloid fibroblasts derived from patients 2, 3, and 6 were transfected with siRNA against Notch1. Only patients 2, 3, and 6 were chosen because only their cells were enough to perform all the experiments. Knockdown of Notch1 with siRNA significantly down-regulated increased levels of NF-κB, NLRP3, ASC, and an intermediate form of caspase-1 in keloid fibroblasts (Figure 4A,B). Depletion of Notch1 in keloid fibroblasts also suppressed levels of α-SMA and TGF- β3 (Figure 4A,B). These results indicate that NLRP3 inflammasome formation and myofibroblast differentiation are enhanced by Notch1 in keloid fibroblasts, suggesting that abnormal inflammatory response and accelerated myoblast differentiation observed in keloid tissue could be improved by reducing Notch1 levels in keloid.

### 2.6. Treatment of Keloid Fibroblasts with Rapamycin Reduces Increased Levels of Inflammasomes and TGF- β3 Proteins

**Figure 5:**
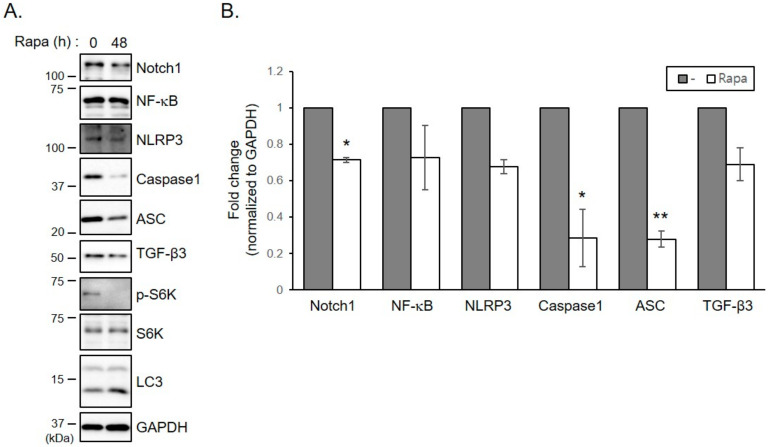
Treatment of keloid fibroblasts with rapamycin reduces increased levels of inflammasome proteins and TGF- β3. Keloid fibroblasts from patients 2, 3, and 6 were seeded and incubated with 0.1 µM of rapamycin (Rapa). Forty-eight hours later, cells were lysed and subjected to immunoblotting with indicated antibodies. (**A**) Gels are representative of five experiments using keloid 2. (**B**) Graphs represent means ± s.d. of five experiments. * *p* < 0.05; ** *p* < 0.01.

We used a strategy to decrease Notch1 levels in keloid fibroblasts by increasing autophagy activity. Rapamycin, a well-known enhancer of autophagy, significantly decreased the increase of Notch1 levels in keloid fibroblasts (Figure 5A,B). Rapamycin treatment also significantly suppressed the increase of inflammasome proteins ASC and caspase-1 levels. Expression levels of NLRP3, NF-κB and TGF-β3 tended to decrease in response to rapamycin treatment, although such decreases were not statistically significant (Figure 5A,B). These results suggest that reducing Notch1 expression levels by increasing autophagic activity which is reduced in keloid can suppress the increase in inflammasome formation and myofibroblast differentiation in keloid fibroblasts.

## 3. Discussion

**Figure 6:**
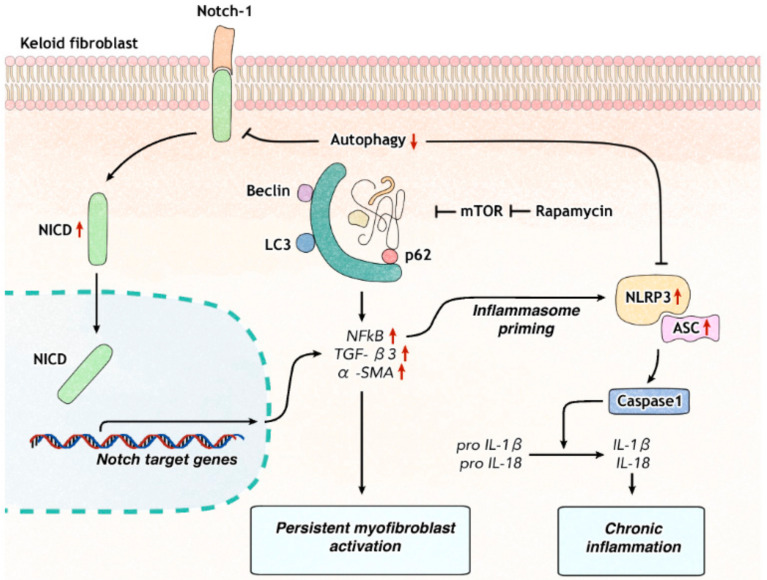
Model for roles of Notch signaling in keloid formation. Autophagy regulates degradation of Notch-1 in dermal fibroblasts. Decreased autophagy in keloid fibroblasts increases the level of Notch intracellular domain (NICD) and enhances the transcription of Notch target genes such as NF-κB, which primes NLRP3 inflammasome proteins and subsequent inflammatory cascade. Notch1 inhibition can suppress NLRP3 inflammasome activation and fibrosis. Rapamycin induces autophagy in keloid fibroblasts, which result in decreased Notch1, NLRP3 inflammasome proteins, and TGF-β3.

Notch1 remains active in keloid fibroblasts, particularly in patients who have pruritus or a recent history of keloid-growth. On the contrary, autophagy is decreased in keloid fibroblasts. Decreased autophagic activity is known to result in an increase of Notch1 [13]. We examined how Notch signaling affected the activation of inflammasomes and myofibroblast differentiation in keloid fibroblasts. We found that Notch1 inhibition regulated innate immune response by down-regulating NLRP3 inflammasome activation and the differentiation into myofibroblasts. Rapamycin, an autophagy enhancer, inhibited Notch1, NLRP3 inflammasome proteins, and myofibroblast marker. This suggests that reduced autophagy plays an important role in the development of chronic inflammatory environment and exaggerated fibrosis via the Notch1-NLRP3 inflammasome signaling pathway in keloid (Figure 6).

Autophagy is a master regulator for the removal of any cellular proteins and organelles as well as alarming signals such as damage-associated molecular pattern (DAMP), pathogen-associated molecular pattern (PAMP), and reactive oxygen species. Emerging evidence supports that autophagy can regulate cardiac fibrosis after myocardial infarction and renal fibrosis [20,21,22]. A recent study has demonstrated that autophagy can decrease tubulointerstitial fibrosis by blocking NLRP3 inflammasome signaling pathway and TGF-β [22]. Increased DAMP and PAMP signals by autophagy disturbance enable NLRP3 inflammasome activation which sequentially secretes pro-inflammatory cytokines IL-1 and IL-18 [23]. Prolonged inflammation can accelerate the transformation of fibroblasts into myofibroblasts [24].

Autophagy regulates Notch degradation and modulates stem cell development and differentiation into neurogenesis [13]. Rapamycin, an mTOR inhibitor, is known to have therapeutic efficacy in keloid [25]. Regarding its therapeutic mechanisms, down-regulation of PCNA (proliferating cell nuclear antigen) and cyclin D1 as mTOR targeted genes has been suggested [25]. Results of the present study suggest that induction of autophagy with rapamycin can attenuate keloidal fibrosis by down-regulating Notch1 and NLRP3 inflammasome in keloid fibroblasts and reduce myofibroblast markers. 

All the while, reduced autophagy can prevent differentiation of hepatic stellate cells into myofibroblast-like cells in liver fibrosis models, leading to reduced fibrogenesis [20]. The regulatory mechanism of autophagy for apoptosis and fibrosis of fibroblasts seems to be complex and different depending on the tissue. 

The role of inflammasome in both normal and keloid fibroblasts remains unclear. Results of the present study suggest that NLRP3 inflammasome is activated in keloid fibroblasts and involved in chronic inflammatory process. NLRP3 inflammasome is often stimulated by high mobility group box 1 (HMGB1), Toll-like receptor (TLR4), NF-κB, and reactive oxygen species known to be increased in keloid [26,27]. One may speculate that increased priming molecules of NLRP3 are due to decreased autophagy. 

NLRP3-dependent IL-1β release may aggravate tissue damage, prolong inflammatory responses, and adversely affect remodeling by inducing continuous myofibroblast differentiation in keloid. Activation of Notch and NLRP3 inflammasome signaling has been reported in hidradenitis suppurativa, a chronic inflammatory condition that is accompanied by severe tissue destruction and scarring [28,29]. Although an increase of *IL-1β* mRNA was observed in keloid fibroblasts, it was hard to detect IL-1β and IL-18 expression levels in keloid fibroblasts in the present study. This might be because fibroblasts do not release cytokines as much as immune cells. Consistently, keloid fibroblasts alone did not express IL-1β or IL-18, although they strongly expressed IL-18 when they were cocultured with keloid keratinocytes, especially under hypoxia culture condition [30]. 

NF-κB signaling can stimulate the transcription of various pro-inflammatory cytokines and proliferation-related molecules by itself or through other signaling pathway including NLRP3 inflammasome. NF-κB, and Signal transducer and activator of transcription are known to contribute to the initiation of inflammation and fibrosis [31]. NF-κB and other proinflammatory cytokines genes are Notch-targeted genes that can start transcription by Notch activation in immune cells [32,33]. In the present study, NF-κB production increased in keloid fibroblasts but decreased by Notch1 silencing. Thus, anti-inflammatory treatment targeting Notch1 could be used to reduce extensive fibrosis in keloid.

TGF-β/Smad are Notch-targeted genes. Their transcription is affected by Notch activation in keloid fibroblasts [26]. TGF-β1 contributes to collagen production and extreme fibrosis in keloid. TGF-β3 has been suggested to play an anti-fibrotic role in fibrogenesis [34]. However, TGF-β3 is involved in collagen synthesis in a TGF-β1-dependent or -independent manner [34]. Recent studies have suggested that blocking both TGF-β1 and TGF-β3 can lead to better improvement of fibrosis in systemic sclerosis than blocking only TGF-β1 [35]. The expression status of TGF-β3 in keloid is not well-known. Since TGF-β1 and TGF-β3 have significant homology and act through the same receptor complex, TGF-β3 may have a pro-fibrotic effect like TGF-β1 in keloid [36]. Overexpression of TGF-β3 was observed in keloid fibroblasts than in normal fibroblasts and the expressive patterns of TGF-β3 with/or without Notch1 inhibition were the same as that of α-SMA in the present study. This suggests that TGF-β3 also plays a role in myofibroblast activation and fibrosis in keloid.

Interaction between Notch and NLRP3 inflammasomes is not clearly known yet. Recently, it has been shown that Notch silencing can worsen acetaminophen-induced liver damage by activating NLRP3 inflammasome through up-regulating HMGB1/TLR4/NF-κB in mice models [37]. In the present study, Notch1 blocked the NLRP3 inflammasome pathway possibly through NF-κB signaling. Further studies are needed to elucidate mechanisms of interaction between Notch and NPRP3 inflammasomes.

In summary, upregulation of Notch1 by autophagy disturbance is involved in the pathogenic and persistent myofibroblast activation and chronic inflammatory condition related to the NLRP3 inflammasome in keloid fibroblasts. Notch1 inhibition down-regulated the NLRP3 inflammasome and α-SMA in keloid fibroblasts. Our results suggest a mechanism linking autophagy to inflammation and myofibroblastic differentiation in keloid fibroblasts induced by the Notch/NLRP3 signaling pathway. Autophagy enhancers and Notch1 inhibitors can be potential treatment targets of keloid by suppressing both NLRP3 inflammasome-induced exaggerated inflammation and scarring.

## 4. Materials and Methods

### 4.1. Patients and Samples

Patients over 18 years old, who visited our clinic at the Eunpyeong St. Mary’s Hospital and had a surgical excision for keloid in the ear between 2016 and 2018 were included in this study. To avoid differences in gene expression due to lesion locations, keloids located in the same location were to be compared. During this period, most lesions in patients undergoing surgical resection were located in the ear, and only keloids in the ear were selected and analyzed in this study. Patients, who did not receive any treatment within 1-month prior to surgical excision were enrolled. This study was approved by the Institutional Review Board of the Catholic University of Korea, Eunpyeong St. Mary’s Hospital (PC17SISI0010) on 14-December-2017. Samples of human keloid fibroblasts were collected from six patients with ear keloid undergoing surgical resection. Table 1 lists the patients’ clinical data. All human keloid samples were taken after obtaining written consent from patients. All data were analyzed anonymously. This study was performed following principles of the Declaration of Helsinki 1975 (revised in 2008).

### 4.2. Cell Culture

An in vitro model of keloid was established by culturing primary keloid fibroblasts from patients with ear keloids. Normal fibroblast cells were obtained from American Type Culture Collection (Normal 1: PCS-201-012) and Promo (Normal 2: statC-12352). Only cells under the 7th passage were used for experiments. Fibroblasts were seeded at a density of 2 × 10^5^ cells per well into 6-well culture plates and cultured in Dulbecco Modified Eagle Medium (DMEM, Gibco BRL, Life Technology, Darmstadt, Germany) supplemented with 10% fetal bovine serum (FBS; Gibco BRL, Life Technology, Darmstadt, Germany) and 1% penicillin/streptomycin (Gibco BRL, Life Technology, Darmstadt, Germany). 

### 4.3. Quantitative Real-Time PCR (qRT-PCR)

Total RNAs were extracted from human keloid and normal fibroblasts using WelPrep Total RNA Prep Kit (WelGene, Republic of Korea). cDNAs were synthesized using an iScript cDNA synthesis kit (Bio-RAD, Hercules, CA, USA) according to the manufacturer’s instructions. These synthesized cDNAs were used for qRT-PCR with TOPreal™ qPCR 2X PreMIX (SYBR Green) (Enzynomics, Republic of Korea) on an CFX Connect^TM^ Real-Time System (Bio-Rad, Hercules, CA, USA). Primer sequences used are as follows: *IL-1β* Forward: 5′-GCCAATCTTCATTGCTCAAGT-3′, *IL-1β* Reverse: 5′-ACTTCATCTGTTTAGGGCCA3′; *IL-18* Forward: 5′-AGCTGAAGATGATGAAAACCTG-3′, *IL-18* Reverse: 5′-ATAGAGGCCGATTTCCTTGG-3′; *GAPDH* Forward: 5′-AGGTCGGAGTCAACGGATTT-3′, GAPDH Reverse: 5′-TGACGGTGCCATGGAATTTG-3′.

### 4.4. Western Blot Analysis

Cells were harvested and lysed with 2× Laemmli sample buffer (4% SDS, 4% β-mercaptoethanol, 20% glycerol, 0.004% bromophenol blue, 0.125M Tris HCl, pH 6.8). Samples were denatured at 95 °C for 10 min and 30 μg of proteins per well was separated using sodium dodecyl sulfate polyacrylamide gel electrophoresis (SDS-PAGE). Proteins were then transferred to nitrocellulose blotting membranes (GE Healthcare-cytiva, Marlborough, MA, USA). These membranes were blocked with 5% skim milk in Tris-buffered saline with 0.05% tween-20 (TBS-T) and subsequently incubated with primary antibodies against Notch1 (D1E11, rabbit, Cell Signaling, Boston, MA, USA), LC3 (Polyclonal, rabbit, Sigma, St. Louis, MO, USA), GAPDH (6C5, mouse, Santa Cruz, Dallas, TX, USA), α-SMA (1A4, mouse, Santa Cruz, Dallas, TX, USA), TGF-β3 (V, rabbit, Santa Cruz, Dallas, TX, USA), p62 (3, mouse, BD Biosciences, San Jose, CA, USA), Beclin1 (D40C5, rabbit, Cell Signaling, Boston, MA, USA), NF-κB (polyclonal, Rabbit, Santa Cruz, Dallas, TX, USA), NLRP3 (Polyclonal, Rabbit, Novus, Littleton, CO, USA), Caspase1 (14F468, Mouse, Novus, Littleton, CO, USA), ASC (B-5, Mouse, Santa cruz, Dallas, TX, USA), p-S6K (Polyclonal, Rabbit, Cell Signaling, Boston, MA, USA), and S6K (Polyclonal, Rabbit, Sino Biological, China). After incubating with a horseradish-peroxidase-conjugated secondary antibody (Santa Cruz, Dallas, TX, USA), specific immunoreactive bands were detected with an enhanced chemiluminescence (ECL) detection system (GE Healthcare-cytiva, Marlborough, MA, USA). Images were obtained and processed with a ChemiDoc XRS+ system and Image Lab software (Bio-Rad, Hercules, CA, USA).

### 4.5. siRNA Transfection

siRNAs against Notch1 (5′-GAACGGGGCUAACAAAGAU-3′) were purchased from Dharmacon (Lafayette, CO, USA). Control siRNAs were purchased from Ambion (Austin, TX, USA). siRNAs (40 nM) were transfected using lipofectamine RNAiMAX (Thermo Fisher Scientific, Waltham, MA, USA) and OptiMEM (Gibco BRL, Life Technology, Darmstadt, Germany) according to the manufacturer’s instructions. After four days of transfection, cells were harvested and analyzed by western blotting.

### 4.6. Drug Treatment

Normal and keloid fibroblast cells were seeded into 24-well plates before drug treatment. Bafilomycin A1 (19-148, EMD Millipore, Burlington, MA, USA) and rapamycin (sc-3504, Santa Cruz, Dallas, TX, USA) were dissolved in dimethyl sulfoxide (DMSO) to prepare stock solution of 100 mM. Bafilomycin A1 (0.4 µM) treatment was performed for 16 h and rapamycin (0.1 µM) treatment was performed for 48 h.

### 4.7. Statistical Analysis

All statistical analyses were carried out using Microsoft Excel (Office 365, Microsoft, Redmond, WA, USA). Student’s t-tests were used to evaluate significant difference between groups. Statistical significance was considered when *p*-value was less than 0.05.

## 5. Conclusions

Our study demonstrated that Notch1 acts as a novel activator of the NLRP3 inflammasome signaling leading to chronic tissue damage and myofibroblast differentiation in keloid progression. Autophagy deficiency resulted in upregulation of Notch1, which leads to the pathogenic and persistent myofibroblast activation and NLRP3 inflammasome-induced exaggerated inflammation in keloid fibroblasts. Treatment with autophagy enhancers or Notch1 inhibitors down-regulated the NRLP3 inflammasome and α-SMA in keloid fibroblasts. Autophagy enhancers or Notch1 inhibitors can be considered as potential therapeutic targets of keloid and further studies are needed in the future.

## Figures and Tables

**Table 1:** Clinical characteristics of patients with keloid.

	Keloid1	Keloid2	Keloid3	Keloid4	Keloid5	Keloid6
**Gender/age**	F/26	F/25	F/65	F/20	F/18	F/23
**Disease duration**	1 yr	2 yrs	3 yrs	3 yrs	1 yr	2 yrs
**Symptoms**	(−)	Pain, pruritus Size increasing	Pain, pruritus Size increasing	(−)	Pain, pruritus Size increasing	(−)
**Clinical findings**	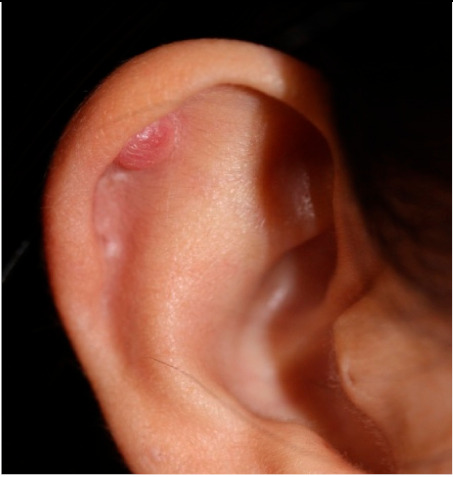	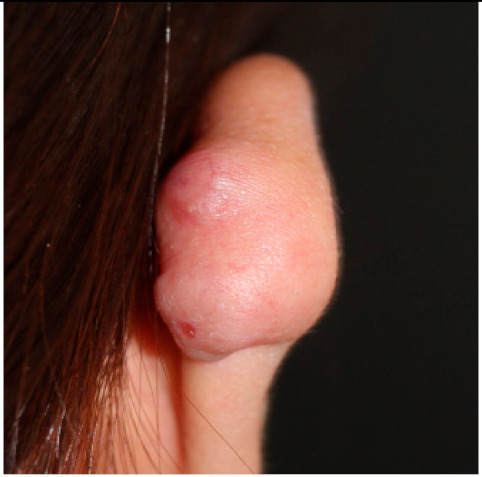	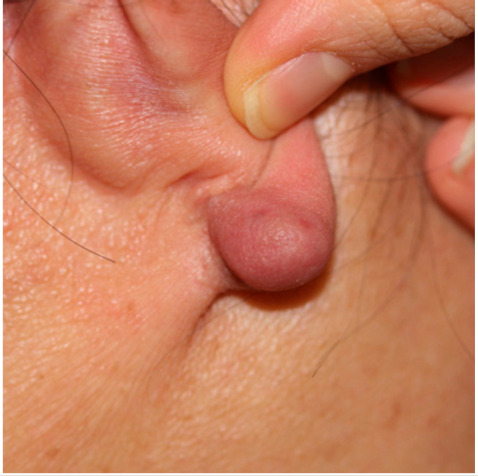	Not available	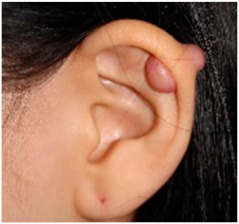	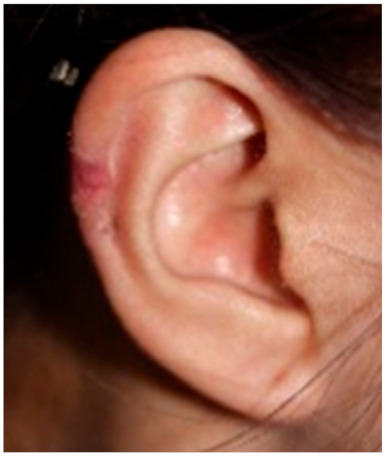
**Previous treatment**	No	ILI 1 month before excision	ILI 4 months before excision	No	No	No

ILI: intralesional corticosteroid injection.

## Abbreviations

ASC	Apoptosis-associated speck-like protein containing a CARD
α-SMA	Alpha-smooth muscle actin
DAMP	Damage-associated molecular pattern
ECM	Extracellular matrix
HMGB1	High mobility group box 1
IL	Interleukin
mTOR	Mammalian target of rapamycin
NF-κB	Nuclear factor kappa B
NICD	Notch intracellular domain
NLRP3	NACHT, LRR, and PYD domains-containing protein 3
PAMP	Pathogen-associated molecular pattern
PCNA	Proliferating cell nuclear antigen
qRT-PCR	Quantitative real-time polymerase chain reaction
TGF-β	Transforming growth factor beta
TLR	Toll-like receptor

## References

[1] Ogawa R. (2017). Keloid and hypertrophic scars are the result of chronic inflammation in the reticular dermis. Int. J. Mol. Sci..

[2] Berman B., Maderal A., Raphael B. (2017). Keloids and Hypertrophic Scars: Pathophysiology, Classification, and Treatment. Dermatol. Surg..

[3] Kim J.-E., Lee J.-H., Jeong K.H., Kim G.M., Kang H. (2014). Notch intracellular domain expression in various skin fibroproliferative diseases. Ann. Dermatol..

[4] Syed F., Hernandez R.M. (2012). Notch signaling pathway in keloid disease: Enhanced fibroblast activity in a Jagged-1 peptide-dependent manner in lesional vs. extralesional fibroblasts. Wound Repair Regen..

[5] Kavian N., Servettaz A., Weill B., Batteux F. (2012). New insights into the mechanism of notch signalling in fibrosis. Open Rheumatol. J..

[6] Edeling M., Ragi G., Huang S., Pavenstädt H., Susztak K. (2016). Developmental signalling pathways in renal fibrosis: The roles of Notch, Wnt and Hedgehog. Nat. Rev. Nephrol..

[7] Dees C., Tomcik M., Zerr P., Akhmetshina A., Horn A., Palumbo K., Beyer C., Zwerina J., Distler O., Schett G. (2011). Notch signalling regulates fibroblast activation and collagen release in systemic sclerosis. Ann. Rheum. Dis..

[8] Aoyagi-Ikeda K., Maeno T., Matsui H., Ueno M., Hara K., Aoki Y., Aoki F., Shimizu T., Doi H., Kawai-Kowase K. (2011). Notch induces myofibroblast differentiation of alveolar epithelial cells via transforming growth factor-{beta}-Smad3 pathway. Am. J. Respir. Cell Mol. Biol..

[9] Jun J.-I., Lau L.F. (2010). Cellular senescence controls fibrosis in wound healing. Aging.

[10] Boya P., Reggiori F., Codogno P. (2013). Emerging regulation and functions of autophagy. Nat. Cell Biol..

[11] Park H., Kang J.-H., Lee S. (2020). Autophagy in neurodegenerative diseases: A hunter for aggregates. Int. J. Mol. Sci..

[12] Mizushima N., Komatsu M. (2011). Autophagy: Renovation of cells and tissues. Cell.

[13] Wu X., Fleming A., Ricketts T., Pavel M., Virgin H., Menzies F.M., Rubinsztein D.C. (2016). Autophagy regulates notch degradation and modulates stem cell development and neurogenesis. Nat. Commun..

[14] Rybstein M.D., Bravo-San Pedro J.M.B.-S., Kroemer G., Galluzzi L. (2018). The autophagic network and cancer. Nat. Cell Biol..

[15] Elliott E.I., Sutterwala F.S. (2015). Initiation and perpetuation of NLRP3 inflammasome activation and assembly. Immunol. Rev..

[16] Ito H., Kanbe A., Sakai H., Seishima M. (2017). Activation of NLRP3 signaling accelerates skin wound healing. Exp. Dermatol..

[17] Lv Z., Wang Y., Liu Y.J., Mao Y.F., Dong W.-W., Ding Z.-N., Meng G.-X., Jiang L., Zhu X.-Y. (2018). NLRP3 inflammasome activation contributes to mechanical stretch–induced endothelial-mesenchymal transition and pulmonary fibrosis. Crit. Care Med..

[18] Torp M.K., Yang K., Ranheim T., Huso Lauritzen K., Alfsnes K., Vinge L.E., Aukrust P., Stenslokken K.O., Yndestad A., Sandanger O. (2019). Mammalian target of rapamycin (mTOR) and the proteasome attenuates IL-1beta expression in primary mouse cardiac fibroblasts. Front. Immunol..

[19] Pan X.-C., Liu Y., Cen Y.-Y., Xiong Y.-L., Li J.-M., Ding Y.-Y., Tong Y.-F., Liu T., Chen X.-H., Zhang H.-G. (2019). Dual role of triptolide in interrupting the NLRP3 inflammasome pathway to attenuate cardiac fibrosis. Int. J. Mol. Sci..

[20] Bernard M., Dieudé M., Yang B., Hamelin K., Underwood K., Hébert M.-J. (2014). Autophagy fosters myofibroblast differentiation through MTORC2 activation and downstream upregulation of CTGF. Autophagy.

[21] Zhang C., Li W., Wen J., Yang Z. (2017). Autophagy is involved in mouse kidney development and podocyte differentiation regulated by Notch signalling. J. Cell. Mol. Med..

[22] Nam S.A., Kim W.-Y., Kim J.W., Park S.H., Kim H.L., Lee M.-S., Komatsu M., Ha H., Lim J.H., Park C.W. (2019). Autophagy attenuates tubulointerstital fibrosis through regulating transforming growth factor-β and NLRP3 inflammasome signaling pathway. Cell Death Dis..

[23] Seveau S., Turner J., Gavrilin M.A., Torrelles J.B., Hall-Stoodley L., Yount J.S., Amer A.O. (2018). Checks and balances between autophagy and inflammasomes during infection. J. Mol. Biol..

[24] El Ayadi A., Jay J.W., Prasai A. (2020). Current approaches targeting the wound healing phases to attenuate fibrosis and scarring. Int. J. Mol. Sci..

[25] Ong C.T., Khoo Y.T., Mukhopadhyay A., Do D.V., Lim I.J., Aalami O., Phan T.T. (2007). mTOR as a potential therapeutic target for treatment of keloids and excessive scars. Exp. Dermatol..

[26] Lei R., Li J., Liu F., Li W., Zhang S., Wang Y., Chu X., Xu J. (2019). HIF-1alpha promotes the keloid development through the activation of TGF-beta/Smad and TLR4/MyD88/NF-kappaB pathways. Cell Cycle.

[27] Luo L., Li J., Liu H., Jian X., Zou Q., Zhao Q., Le Q., Chen H., Gao X., He C. (2017). Adiponectin is involved in connective tissue growth factor-induced proliferation, migration and overproduction of the extracellular matrix in keloid fibroblasts. Int. J. Mol. Sci..

[28] Frings V.G., Sennefelder H., Presser D., Goebeler M., Schmidt M. (2019). Altered NOX expression does not seem to account for epidermal NLRP 3 inflammasome activation in hidradenitis suppurativa. Br. J. Dermatol..

[29] Marasca C., Scala E., Di Caprio R., Raimondo A., Cacciapuoti S., Balato N., Fabbrocini G. (2019). Notch dysregulation and hidradenitis suppurativa, psoriasis, atopic dermatitis and lichen planus: Let’s talk about Numb. Br. J. Dermatol..

[30] Do D., Ong C., Khoo Y., Carbone A., Lim C., Wang S., Mukhopadhyay A., Cao X., Cho D., Wei X. (2012). Interleukin-18 system plays an important role in keloid pathogenesis via epithelial-mesenchymal interactions. Br. J. Dermatol..

[31] Hou J., Ma T., Cao H., Chen Y., Wang C., Chen X., Xiang Z., Han X. (2017). TNF-α-induced NF-κB activation promotes myofibroblast differentiation of LR-MSCs and exacerbates bleomycin-induced pulmonary fibrosis. J. Cell. Physiol..

[32] Espinosa L., Cathelin S., D’Altri T., Trimarchi T., Statnikov A., Guiu J., Rodilla V., Inglés-Esteve J., Nomdedeu J., Bellosillo B. (2010). The Notch/Hes1 pathway sustains NF-κB activation through CYLD repression in T cell Leukemia. Cancer Cell.

[33] Xiu Y., Dong Q., Fu L., Bossler A., Tang X., Boyce B., Borcherding N., Leidinger M., Sardina J.L., Xue H.-H. (2020). Coactivation of NF-κB and Notch signaling is sufficient to induce B-cell transformation and enables B-myeloid conversion. Blood.

[34] Murata H., Zhou L., Ochoa S., Hasan A., Badiavas E., Falanga V. (1997). TGF-beta3 stimulates and regulates collagen synthesis through TGF-beta1-dependent and independent mechanisms. J. Investig. Dermatol..

[35] Komai T., Okamura T., Inoue M., Yamamoto K., Fujio K. (2018). Reevaluation of pluripotent Cytokine TGF-β3 in Immunity. Int. J. Mol. Sci..

[36] Lichtman M.K., Otero-Viñas M., Falanga V. (2016). Transforming growth factor beta (TGF-β) isoforms in wound healing and fibrosis. Wound Repair Regen..

[37] Jiang L., Ke M., Yue S., Xiao W., Yan Y., Deng X., Ying Q.-L., Li J., Ke B. (2017). Blockade of notch signaling promotes acetaminophen-induced liver injury. Immunol. Res..

